# Our Advertisers

**Published:** 1878-05

**Authors:** 


### OUR ADVERTISERS.

We present to our readers, in each issue of our JOURNAL, a few reliable advertisements. They are selected with care from the number presented, and we try to have them the best of their kind. If we are at any time deceived, we do not hesitate to cancel our contract and refuse to continue them. We call your attention to the following:

In speaking of LACTOPEPTINE in our last it was called Loctopepsin, which mistake was overlooked in reading the proof. The large sales of this important remedy indicate the favor in which it is held by the profession. We have had but little experience in its use, but a friend informs us that he has found it excellent in many cases of indigestion and dyspepsia.

TROMMER'S EXTRACT OF MALT is not only pure and reliable, but is warranted to keep for years in any climate. We have found much benefit to accrue from its use in debility, and we refer to Art. III. this number for its application in Phthisis.

BLOOD TONIC as manufactured by Caswell, Hazard & Co., is not a mere tonic, but claims to contain blood-making, and life-sustaining properties, calculated to support the system under wasting diseases. It is entirely free from any drug.

WYETH'S DIALYSED IRON is well known from its action as an antidote to arsenic. Although some chemists theoretically disbelieve in its efficiency, still the cases reported are proof of its value. John Wyeth & Bro. are also to be commended for their fine compressed pills, which are prepared without excipient or coating, and are more easily swallowed than any other form of pill.

To those in need of a reliable and pleasant form of administering drugs, MC-KESSON & ROBBIN'S Granules, will be found to fulfill the want. They are made with great care and exactness, and may be depended upon for unvarying action.

SCOTT'S Emulsion of Cod Liver Oil with Hypophosphites, remains permanent even in extreme hot weather, and is very palatable in comparison with many others which we have tested.

SHEPARD & DUDLEY'S Instruments may be found in stock at King's Drug Store, while C. M. Lyman keeps a full line of Tieman's excellent goods.

